# Screening and Scoring of Antimicrobial and Biological Activities of Italian Vulnerary Plants against Major Oral Pathogenic Bacteria

**DOI:** 10.1155/2013/316280

**Published:** 2013-11-04

**Authors:** Gianmaria F. Ferrazzano, Lia Roberto, Maria Rosaria Catania, Angela Chiaviello, Antonino De Natale, Emanuela Roscetto, Gabriele Pinto, Antonino Pollio, Aniello Ingenito, Giuseppe Palumbo

**Affiliations:** ^1^Dipartimento di Neuroscienze e Scienze Riproduttive ed Odontostomatologiche, Zona Ospedaliera, Via Pansini 5, 80131 Napoli, Italy; ^2^Dipartimento di Medicina Molecolare e Biotecnologie Mediche, Zona Ospedaliera, Via Pansini 5, 80131 Napoli, Italy; ^3^Dipartimento di Biologia, Complesso Universitario di Monte Sant'Angelo, Via Cintia 21, 80126 Napoli, Italy

## Abstract

This study aims to evaluate the activity of Italian vulnerary plants against the most important oral pathogenic bacteria. This estimate was accomplished through a fivefold process: (a) a review of ethnobotanical and microbiological data concerning the Italian vulnerary plants; (b) the development of a scoring system to rank the plants; (c) the comparative assessment of microbiological properties; (d) the assessment of potential cytotoxic effects on keratinocyte-like cells and gingival fibroblasts in culture by XTT cell viability assay; (e) clinical evaluation of the most suitable plant extract as antibacterial agent in a home-made mouthwash. The study assays hexane (H), ethanol (E), and water (W) extracts from 72 plants. The agar diffusion method was used to evaluate the activity against *Streptococcus mutans, Streptococcus sobrinus*, *Lactobacillus casei,* and *Actinomyces viscosus*. Twenty-two plants showed appreciable activity. The extracts showing the strongest antibacterial power were those from *Cotinus coggygria* Scop., *Equisetum hyemale* L., *Helichrysum litoreum* Guss, *Juniperus communis* L., and *Phyllitis scolopendrium* (L.) Newman subsp. *scolopendrium*. The potential cytotoxic effect of these extracts was assessed. On the basis of these observations, a mouth-rinse containing the ethanolic extract of *H. litoreum* has been tested *in vivo*, resulting in reduction of the salivary concentration of *S. mutans*.

## 1. Introduction

Dental caries is one of the most prevalent chronic diseases of people worldwide. Despite it recognizes multifactorial etiology, this illness is fundamentally a bacterial infectious disease [1].

A wide group of microorganisms have been isolated from dental lesions, including *Streptococcus mutans, Streptococcus sobrinus*, *Lactobacillus casei, *and *Actinomyces viscosus*, that all play a significant role in the mechanism of caries formation. These bacteria are indeed the main pathogens involved in the initiation of its development [2]. The use of antibiotics for prevention of dental caries has been fully investigated. Large spectra antibiotics, orally or systemically administered for the prevention of caries, tend to suppress the resident bacterial population thus facilitating the overgrowth of opportunistic pathogens such as *Candida albicans*. Moreover, they may enter the oral cavity via saliva and gingival crevicular fluid and lead to a negative imbalance in the oral microbiota [3]. A further well-recognized drawback is a direct consequence of the past/current misuse of antibiotics that has determined a progressive resistance of bacteria with a consequent loss of therapeutic efficacy [4]. Other reasons that limit the use of conventional antibiotic therapy for the eradication of cariogenic bacteria stand on the presence of a barrier effect caused by the bacterial glycocalyces. Indeed, these polysaccharides have been implicated in providing a protective structure, since bacteria in the adherent or sessile mode of growth demonstrated increased resistance to antibiotics and to host humoral and cellular immune responses [5]. Therefore, as the current therapeutic strategies to prevent dental diseases are not fully void of side effects, the development of novel and alternative approaches for microbial control should be considered not only advantageous but also necessary.

To now, numerous plant products have been investigated for their effectiveness in the prevention of dental plaque formation, but only a very small number of these natural products has found therapeutic application. The reasons of such limited use stand on various factors as adequate effectiveness, stability, smell, taste, and, not last, cost [6].

Vulnerary plants, that is, “wound healing plants”, represent one of the largest legacies provided by folk medicine worldwide [7].

The therapeutic activities of these plants are numerous and span widely: some present astringent properties, other are endowed of anti-inflammatory power, and some may be immunestimulant and/or possess recognized antimicrobial activity [8]. For these reasons, vulnerary plants represent a treasure house for searching of such type of compounds, especially those aimed at fighting microbial infections. Indeed, Brantner and Grein [9] demonstrated that about 60% of plant extracts used in traditional medicine exhibited antibacterial action.

This study was planned as a first large screening on vulnerary plants growing in Italy and is aimed at selecting in particular those extracts that potentially could control the whole oral health acting against the cariogenic bacteria while simultaneously favoring the healing of gingival and other oral lesions [10].

The first part of this work consisted in the choice and selection of collected plants on the basis of a definite scoring system. The successive steps were performed in the laboratory and comprised the extraction procedures with solvents of different hydrophobicity (Hexane, ethanol, and water); the evaluation of individual antimicrobial activity against selected bacterial strains (*Streptococcus sobrinus, S*. *mutans, Lactobacillus casei, *and *Actinomyces viscosus*), and the assessment of potential cytotoxic or growth-stimulating properties (of extracts endowed of antimicrobial activity) on two epithelial cell lines. Finally, a pilot *in vivo* experiment was undertaken with the aim to examine the antimicrobial efficacy of an experimental mouth-rinse prepared with *Helichrysum litoreum *ethanol extracts (HEE), in order to reduce *Streptococcus mutans *levels in saliva.

## 2. Methods and Materials

### 2.1. Ranking Procedure

Seventy-two plants, reported as vulnerary in at least three different ethnobotanical records, were ranked according to the following criteria.

- Indication of an established vulnerary use in traditional medicine of different countries:
  - extensively used: 3 points,
  - used: 2 points,
  - occasionally used: 1 point.
- Specific use in the treatment of oral affections:
  - extensively used: 3 points,
  - used: 2 points,
  - occasionally used: 1 point.
- Available data on the antimicrobial activity of extracts or isolated principles from a selected plant:
  - frequently reported: 3 points,
  - some times reported: 2 points,
  - rarely reported: 1 points.
- Distribution:
  - plant easily found, forming large population: 1 point,
  - Plant difficult to find or forming undersized populations: 0 points.

### 2.2. Collection of Plant Samples

Plants were collected during spring and summer of 2008 in the regional parks of Matese and Cilento, Campania, Italy, or in the Botanical Garden of University Federico II of Naples. Shortly after collection, plants were oven-dried at 50°C for 48 hours. Dried plants were finely grinded, and the resulting powder-like materials were stored at −20°C. For each plant, a voucher sample was saved at the Department of Biological Sciences, University Federico II of Naples.

### 2.3. Preparation of Plant Extracts

Exactly 4 g of each powdered plant material were soaked in 40 mL of hexane, then in 40 mL of ethanol and finally in 40 mL of water. In general, extraction procedure was carried out at room temperature (~25°C) in 100 mL Erlenmeyer flasks. These were kept in ultrasonic baths for 30 minutes, followed by 24 hours continuous stirring (90 rpm) in a rotary shaker. Extracts were then filtered on paper (Whatman, n.1) and concentrated using a vacuum roto-evaporator at 38°C. The dried material was finally stored at −20°C. Powders were solubilized in aqueous DMSO (10%) before further use. To assess biological activity of epithelial cells, the solutions were diluted 1 : 10 with water (final DMSO concentration was 1%).

### 2.4. Bacterial Strains

The bacterial strains used for the screening were *Actinomyces viscosus *(ATCC 19246) and *Lactobacillus casei *(ATCC 393), obtained from American type Culture Collection (ATCC; Rockville, MD, USA); *Streptococcus mutans *and *Streptococcus sobrinus *were from clinical specimens obtained at the Diagnostic Unit of Microbiology of the University of Naples “Federico II.” Bacteria were grown on Trypticase Soy Agar II with 5% Sheep Blood (TSS; Becton Dickinson, USA) plates at 37°C in 5% CO_2_ for 48 h.

### 2.5. Antimicrobial Tests

The initial screening of antibacterial activity was performed using the agar well-diffusion method. Inocula were prepared from overnight cultures of each bacterial strain and adjusted to 0.5 McFarland standard of turbidity. Bacterial strains were evenly spread on the surface of TSS agar plates using sterile swabs, and three wells of 8 mm diameter were punched into the agar medium. The vacuum-dried extracts from water, ethanol and hexane, were redissolved in water containing 10% DMSO (Sigma Aldrich Milan, Italy); these stocks were serially diluted to give concentrations, referred to the dry powder, ranging from 200 to 12.5 mg/mL. The assay was initiated pouring 100 Kl of each of these solutions into separate wells (100 Kl of 10% DMSO solution were used as negative control). As a positive control, we used Triclosan, a polychloro phenoxy phenolic antibacterial agent widely used as antigingivitis in toothpastes and mouthwashes. Our control solution was constituted by Triclosan (0.3%) in water containing 10% DMSO. The plates were incubated at 37°C in 5% CO_2_ atmosphere for 48 h. The antibacterial activity of plant extracts was evaluated by measuring the diameter (expressed in mm) of inhibition zone observed around each well. All tests have been performed in triplicate and repeated twice.

The minimum inhibitory concentration (MIC) was measured by the standard microdilution method in 96-wells polystyrene plates using Brain-Heart Infusion (BHI) medium. The starting inoculum was 5 × 10^5^ CFU mL^−1^, and the concentrations for the plant extracts ranged from 100 to 6.25 mg mL^−1^ (twofold dilution). The MIC was considered the lowest concentration of extract able to inhibit any visible bacterial growth. To determine the MBC (minimal bactericidal concentration), 50 *μ*L of bacterial suspension from the wells containing extract concentrations equal or higher than the MIC were inoculated in 5 mL of sterile BHI medium and incubated for 24 h at 37°C in 5% CO_2_ atmosphere. MBC was considered the lowest concentration that inhibited completely bacterial growth. Each extract was tested in triplicate; each experiment was performed twice.

### 2.6. Cell Lines

Human gingival fibroblasts (HGF-1) and keratinocyte cell line HaCaT cell lines were both obtained from ATCC (Rockville, MD, USA). Both cell lines were cultured in Dulbecco's Modified Eagle Medium, containing 10% Foetal Calf Serum (FCS), 2 mM L-glutamine, and 50 Kg/mL gentamicin (fibroblasts) or 100 Kg/mL streptomycin + 100 units/mL penicillin (HaCaT). The media were changed every second day. Cell culture reagents were purchased from Life Technologies (San Giuliano Milanese, Italy).

### 2.7. Biologic Assays on Human Normal (Gingival Fibroblasts) or Immortalized (Keratinocyte-Like) Cells

Cell viability was assayed by using the Cell Proliferation Kit II (XTT, Roche, Milan, Italy). This assay is based on the cleavage of the yellow tetrazolium salt XTT to form an orange formazan dye by metabolic active cells [11]; therefore, this conversion only occurs in viable cells. The plant extracts were provided to our laboratory in the relative extraction solvents having a nominal concentration of 50 mg/mL. The extracts were dried under vacuum and redissolved in DMSO 10% in water. Samples were stored at −30°C until use. Before measurements, samples were brought to room temperature under agitation and added to culture media in a ratio 1 to 10. In detail, 90 *μ*L of suspensions of fibroblast or HaCaT cells (containing ~1 × 10^4^ cells in complete medium) were seeded into 96-well plates. Then, 10 *μ*L of each extract (50 mg/mL in 10% DMSO) were added to each well so that the final extract concentration was 5 mg/mL, while the DMSO content was reduced to 1%. Cells were incubated in these conditions at 37°C for 24 hours in 5% CO_2_ atmosphere. Triplicate samples were prepared for any individual condition. As a positive control for cyto-toxicity, we used Triclosan at low concentration (0.03% as compared with 3% used in toothpastes). This synthetic is a polychloro-phenoxy phenolan endowed with antibacterial and antifungal properties. For these reasons, it is currently largely used in oral hygiene as additive of toothpastes to prevent gingivitis.

### 2.8. *In Vivo* Test: Efficacy of *H. litoreum* Ethanolic Extract against *Mutans streptococci*

The study enrolled 28 volunteers (12 males and 16 females) ranging in age from 12 to 18 years. The participants were recruited from young patients of the Department of Paediatric Dentistry University Hospital of Naples “Federico II,” Italy. The study plan was approved by the Local Committee for Medical and Health Research Ethics University of Naples “Federico II.” Patients and their parents received verbal and written explanations about the study and written informed agreement form to be signed to participate. The study protocol was in accordance with the Helsinki Declaration of Human Rights.

Inclusion criteria were good general health (ASA I-II) and agreement to strictly comply with the procedures indicated by the study protocol. Exclusion criteria were prior exposure (less than two weeks) to antibiotic treatment and/or prior use (<12 hours) of antibacterial mouthwashes. Similarly, individuals carrying fixed orthodontic appliances were excluded from this study. Participating volunteers were randomly distributed into two groups of 14 subjects: group A representing patients using *H. litoreum *mouthwash (a 1% ethanolic extract of *H. litoreum*, at a concentration of 12,5 mg/mL), and group B representing those using placebo solution (20 mL of a 1% ethanol in water). The taste of the extracts was slightly unpleasant, but we purposely avoided the addition of flavoring additives to exclude potential interferences.

A first sample of saliva was collected from each patient before the treatment (t0) in order to establish the baseline levels of mutans streptococci. After the collection of first sample, all participants were instructed to mouth-rinse with 20 mL of *H. litoreum *extract (group A) or placebo solution (group B) for 1 minute. This procedure has to be repeated three times a day (after breakfast, after lunch, and at the bed time), after normal oral hygiene procedures, for fourteen consecutive days. Saliva samples were collected at day 7 (t1) and 14 (t2) of treatment. *Mutans streptococci *counts in saliva were determined by using a “Caries risk test,” namely, the CRT bacteria assay by Ivoclar Vivadent, Bologna, Italy, a method used in dental clinics for a semiquantitative evaluation of the main cariogenic bacteria in saliva [12]. The saliva samples were collected in sterile containers and used to wet the blue Mitissalivarius-agar with bacitracin for determination of mutans streptococci as indicated by the kit's manufacturer. Vials were incubated at 37°C for 48 hours.

### 2.9. Statistical Analysis

All data from *in vitro *tests (Section 2.7) were expressed as mean ± SD. Significance was assessed by the Student's *t*-test for unpaired data for comparisons between two means. Statistical significance was defined as *P < 0.01; **P < 0.001; ***P < 0.0001. All data from *in vivo *samples (Section 2.8) were processed with the Statistical Package for Social Sciences (version 10.0, SPSS Inc., Chicago, IL, USA). A regression binary logistic analysis was made. Statistical significance level was established at *P* < 0.05.

## 3. Results

### 3.1. Plant Selection and Ranking

Data from Guarrera [13] and from the ethnobotanical database of Campania and South Italy [14] were used in listing 312 vulnerary plants of Italian flora. Since this number appears to be quite large and not easy to manage, only the species reported as vulnerary in at least three different ethnobotanical reports were selected and included in this study. On this basis, only 72 plants responded to this criterion. These plants were then ranked according to the scoring system outlined in Section 2.  Table 1 resumes the score assigned to each plant associated with the estimated antibacterial activity. The antibacterial potential of the selected plants, assessed by using ethnobotanical data, ranked 20 species with a high score (i.e., ≥6) and the remaining 52 with a lower score (between 5 and 1).

### 3.2. Antimicrobial Activity

Out of 72 plants tested, only 20 (28%) exhibited variable degrees of inhibitory activity against one or more bacterial species (Table 1, last column). The most active plants were all characterized by the highest score, while only few extracts from those with the lower scores were endowed of a measurable antibacterial activity. The only notable exception is represented by *Equisetum hyemale*, which at least at the highest concentration was definitely effective towards three strains, *S. sobrinus*, *S. mutans *and *L*. *casei*.  Table 2 presents the results obtained in typical well-diffusion bioassays, compared to that shown by a Triclosan 0.3% solution. The water extract from *Cotinus coggygria*. engenders the major effects, being active against all the four bacteria at any concentration tested. The hexane extract of *Juniperus communis *inhibited the growth of all bacteria except that of *L. casei*. The ethanolic extract obtained from *Helichrysum litoreum *was effective against *S. mutans *and *A. viscosus, *while the ethanolic extract from *Phyllitis scolopendrium *subsp. *scolopendrium *was successful against *S. mutans *and *L. casei*. The ethanolic extracts of *Bellis perennis *and *Ceterach officinarum *Willd. s.l. showed a small inhibitory activity against *S. sobrinus*; when used at the highest concentrations, both extract induced a tiny reduction in the growth rate of *L. casei*. The ethanolic extracts of *Thymus vulgaris *L. s.l. exhibited a mild activity against *S. sobrinus *and *L. casei*.

A selective inhibitory activity towards *A. viscosus *was evidenced by the ethanolic extract of *Gentiana lutea *L. s.l. The other plant extracts, but only at the highest concentration tested, were all endowed of a scarcely noticeable activity against two or even one bacterial strain. The antimicrobial activity of more effective extracts was investigated also in terms of minimum inhibitory concentration (MIC) and minimum bactericidal concentration (MBC) (Table 3). The water and ethanolic extracts of *Cotinus coggygria *demonstrated a considerable activity against all the bacteria tested, while the extract from hexane appeared very selective against *S. sobrinus*. Similarly, the ethanolic extract of *Helichrysum litoreum *was very effective against *S. mutans *and *A. viscosus*. All the remaining extracts were characterized by high MIC values (≥100 mg/mL).

### 3.3. Activity of Plant Extracts on Human Cells Viability

**Figure 1:**
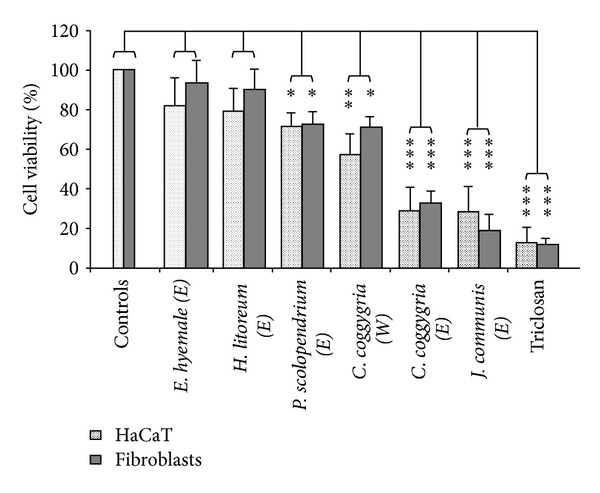
Effects of selected plant extracts (as indicated) on viability of keratonocyte-like cells and gingival fibroblasts as measured by XTT assay. Statistical significance is defined as *P < 0.01; **P < 0.001; ***P < 0.0001.

Activity of the extracts was assessed by observing the consequences of their action on the viability of two eukaryotic cell lines, one normal (human gingival fibroblasts) and one immortalized (HaCaT cells). The assay was done by means of a specific test (XTT assay) that provides information on cell proliferation/impairment through the assessment of changes in mitochondrial specific enzymatic activity of cells under observation. In general, no statistically significant changes were observed in cells treated with extracts from most plants included in this study (extracts from 72 plants). However, some interesting exceptions ensued, as among the extracts endowed of *in vitro* antibacteric activity, some were void and some were endowed of inhibitory properties on the growth of both fibroblasts and HaCaT cells. Specifically, the ethanolic extract from both *H. litoreum *and *E. hyemale *did not affect the viability of both cell lines, while the ethanolic extract from *P. scolopendrium *and the water extract from *C. coggygria *were slightly but measurably inhibitory (*P* < 0.001). The ethanolic extract from *C. coggygria *and the hexane extract from *J. communis *appeared to be, in turn, frankly toxic to both cell lines (*P* < 0.0001) The control of these experiments was provided by Triclosan, whose addition to cells at low concentration (0.03%, i.e., up to 100 times lower than that used in toothpastes) caused a profound reduction (up to >90%) in the cell viability as measured by XTT assay (Figure 1).

### 3.4. Preliminary *In Vivo* Assessment on the Efficacy of *H. litoreum* Ethanolic Extract against *Streptococcus mutans*

**Figure 2:**
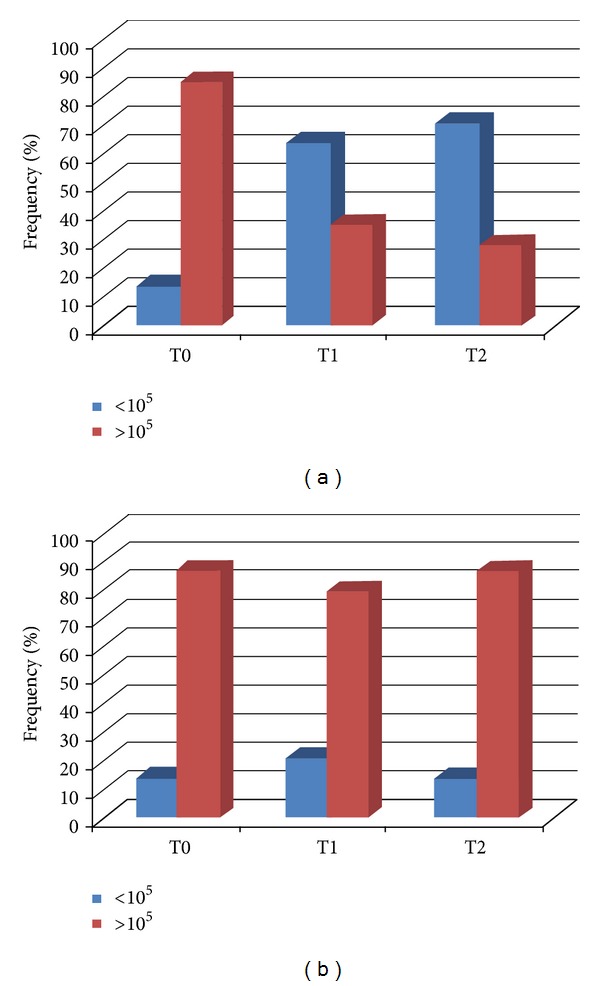
(a) Variation of *S. mutans* concentration (CFU/mL) at t0, t1, and t2 (group A). (b) Variation of *S. mutans* concentration (CFU/mL) at t0, t1, and t2 (group B).

The CRT bacteria assay results were expressed as a low (<10^5^ CFU) or a high (>10^5^ CFU) bacterial count. Variations in *S. mutans *density of the CFU (CFU/mL) at t0, t1, and t2 for the test group (A) are summarized in Figure 2(a). The differences in CFU (CFU/mL) density of MS were statistically significant between t0 and t1 (*P* = 0.012) and between t0 and t2, (*P* = 0.005); between t1 and t2 they were not statistically significant.

Variations in *S. mutans *density of the CFU (CFU/mL) at t0, t1, and t2 for the control group (B) were represented in Figure 2(b). The differences in CFU (CFU/mL) density of *S. mutans *between t0 and t1, t0, and t2, t1 and t2 were not statistically significant. At t0, the differences in CFU (CFU/mL) density of *S. mutans *between groups A and B were not statistically significant, while at t1 and t2 the differences were statistically significant, respectively [t1: OR = 0.15 (CI = 0.28–0.81); t2: OR = 0.06 (CI = 0.01–0.44)].

## 4. Discussion

We observed and reported that about twenty vulnerary plants of Italian flora showed inhibitory activity against cariogenic bacteria. A good correlation was found between the speculative ranking system we adopted and the results of some specific bioassays: taking into account a cut-off value of 6 points, almost all the plants endowed of a measurable activity presented a score above this boundary.

The species demonstrating an antimicrobial action belong to 13 families of vascular plants, not having any phylogenetic relationship. However, the most represented family was that of Lamiaceae, which includes many species with documented biocide activity [15]. All the bacterial strains tested revealed a higher sensitivity to ethanolic extracts, followed by aqueous extracts. While the precise reasons of the higher activity displayed by ethanolic extracts are not clear to now, the possible presence of flavonoids and related compounds (very soluble in alcohols) may explain such property. Indeed, the flavonoids inhibitory action against cariogenic bacteria has been suspected since long time [16]. The aqueous extracts contain more polar compounds [17], which are probably less effective against cariogenic bacteria, due to the strong hydrophobicity of their cell surfaces [18]. To now, the minimal antimicrobial effect of extracts from hexane finds no clear explanation and definitely deserves further investigation. Interesting enough, we did not observe activity in extracts from plants as *Thymus vulgaris, *which, indeed, are known for their antimicrobial action [19]. A possible explanation for such discrepancy may reside in environmental factors and plant chemotypes [20] that can both strongly affect the amount of the active compounds produced by the plant.

*Cotinus coggygria *was the most active species among the plants selected for the screening. This plant is largely used in the Balkan and Anatolian regions to cure wounds and reduce inflammations, as well as for the treatment of gastrointestinal and respiratory disorders [21]. In Asiatic countries, *C. coggygria* is also known as a bactericide and frequently administered against hepatitis and even anemia [22]. A relative of this species, *Rhus coriaria*, which grows in the Mediterranean region, has demonstrated inhibitory properties towards *Streptococcus mutans *and *S. sanguinis*, common components of dental plaque [23]. These authors attributed this effect to the presence of large amounts of tannins in the plant. Tannins can then generate smaller phenolics compounds (pyrogallol, catechol, and ellagic acid) with known bactericidal actions. Similarly, *C. coggygria *is very rich in phenolic compounds [24] and displays a significant antimicrobial activity.

*Helichrysum litoreum *Guss is a species endemic to Central-South Italy, Sicilia, and Sardinia [25]. Preliminary research evidenced bactericidal activity of *H. litoreum *crude extracts, as also reported for other *Helichrysum *species [26]. In the species *H. compactum, *the antimicrobial activity has been attributed to flavonoids and chemically related compound [27]. The data obtained in the present study on the specific activity of *H. litoreum *extracts against *S. mutans *and the absence of cytotoxic effects are in agreement with the results previously reported for *H. italicum *by Nostro et al. [28]. We have found that also *Phyllitis scolopendrium, *a fern belonging to Aspleniaceae, possesses significant activity towards cariogenic bacteria. The same holds true for the other Pteridophyta, *E. hyemale*, a plant of Euro-Asiatic origin, but also diffused in the American continent. Indeed, recently it has been reported a specific activity of this species against *Staphylococcus aureus *[29], but, to date, this is the first report describing inhibitory activity of the plant against oral pathogen. The extracts of *Phyllitis scolopendrium *and *C. coggydria* present a small but measurable effect on cell viability.

Another active species, *J. communis, *is already known for its antimicrobial properties: it has been shown that the essential oil from *J. communis *berries had definite inhibitory effect against Gram-positive and Gram-negative bacterial species [30] and that the hexane extract of  *J. communis *leaves was extremely effective against pathogenic multiresistant bacteria [31]. The extract is measurably cytotoxic.

Interesting enough, two out of six active extracts have shown no statistically significant cytotoxicity on both keratinocyte-like transformed cells and normal gingival fibroblastsas the cells wellbeing was fully unaffected by their presence (even for extended time). At variance, two other extracts were endowed of slight growth-inhibitory properties, and two were frankly cytotoxic (the reduction in cell viability in the latter cases was similar to that caused by the polychloro-phenoxy phenol *Triclosan*, a widely used antibacterial and antifungal agent). The two noncytotoxic extracts, namely, *H. litoreum *and *E. hyemale*, displayed a different antimicrobial activity, the first being clearly more active.

The present *in vivo* study has shown that a regular daily rinsing with mouthwash containing *H. litoreum *ethanol extract could reduce on 50% of subjects the salivary levels of *S. mutans*, which are the most virulent cariogenic pathogens in the oral cavity. This is probably due to both inhibition of growth and adherence of *S. mutans *cells to teeth surfaces.

## 5. Conclusions

The results of this study on a large sample of vulnerary plants of Italian flora have identified a limited number of extracts that may find real application in the prevention of dental caries, as they work as effective weapons against all the major bacterial constituent of the plaque. Further, long-term studies *in vivo* involving more subjects are needed to clarify if this approach could represent an effective complementary strategy for reducing the severity of this illness. 

## Figures and Tables

**Table 1:** Weighing and scoring of 72 vulnerary plants of Italian flora.

	Established vulnerary use	Used against oral affections	Tested antimicrobial activity	Species distribution	Final score	Inhibitory activity against bacteria of oral flora
*Plantago major* L. subsp. *major* (DSB52) Plantaginaceae	2	3	3	1	**9**	−
*Rosmarinus officinalis *L. (DSB56) Lamiaceae	3	2	3	1	**9**	−
*Sambucus nigra *L. (DSB58) Caprifoliaceae	2	3	3	1	**9**	+
*Malva sylvestris* L. subsp. *sylvestris* (DSB45) Malvaceae	2	3	2	1	**8**	−
*Bellis perennis* L. (DSB10) Asteraceae	2	2	2	1	**7**	++
*Cupressus sempervirens* L. (DSB20) Cupressaceae	1	3	2	1	**7**	++
*Juniperus communis* L. (DSB37) Cupressaceae	1	3	2	1	**7**	+++
*Lavandula angustifolia* Mill. subsp. *angustifolia *(DSB39) Lamiaceae	1	2	3	1	**7**	++
*Mentha aquatica* L. s.l. (DSB46) Lamiaceae	2	2	2	1	**7**	−
*Thymus vulgaris* L. s.l. (DSB69) Lamiaceae	1	3	3	0	**7**	++
*Arctium lappa* L. (DSB5) Asteraceae	1	1	3	1	**6**	−
*Ceterach officinarum* Willd. s.l. (DSB17) Aspleniaceae	3	1	1	1	**6**	++
*Cotinus coggygria* Scop. (DSB19) Anacardiaceae	1	2	2	1	**6**	+++
*Equisetum arvense* L. s.l. (DSB25) Equisetaceae	1	2	2	1	**6**	−
*Eucalyptus globulus* Labill. (DSB29) Rutaceae	1	2	2	1	**6**	+
*Gentiana lutea* L. s.l. (DSB32) Gentianaceae	2	2	2	0	**6**	+
*Helichrysum litoreum *Guss (DSB34) Asteraceae	1	1	3	1	**6**	+++
*Oxalis corniculata* L. (DSB48) Oxalidaceae	1	1	3	1	**6**	−
*Rumex crispus* L. (DSB57) Polygonaceae	1	3	1	1	**6**	−
*Verbena officinalis* L. (DSB 72) Verbenaceae	1	2	2	1	**6**	+
*Adiantum capillus-veneris* L. (DSB2) Adiantaceae	0	1	3	1	5	−
*Alliaria petiolata* (M. Bieb.) Cavara et Grande (DSB4) Brassicaceae	3	1	0	1	5	−
*Artemisia annua* L. (DSB6) Asteraceae	0	2	2	1	5	−
*Asphodelus ramosus* L. subsp. *ramosus* (DSB7) Asphodelaceae	1	1	2	1	5	−
*Calamintha nepeta* (L.) Savi subsp. *nepeta* (DSB12) Lamiaceae	1	1	2	1	5	−
*Calystegia silvatica* (Kit.) Griseb. (DSB13) Convolvulaceae	3	1	0	1	5	−
*Cynodon dactylon* (L.) Pers. (DSB22) Poaceae	1	1	2	1	5	−
*Cynoglossum creticum* Mill. (DSB23) Boraginaceae	1	2	1	1	5	+
*Equisetum telmateia* Ehrh. (DSB27) Equisetaceae	1	1	2	1	5	−
*Erigeron canadensis* L. (DSB28) Asteraceae	2	1	1	1	5	−
*Geranium purpureum* Vill. (DSB33) Geraniaceae	2	1	1	1	5	−
*Heliotropium europaeum* L. (DSB35) Boraginaceae	1	2	1	1	5	−
*Hypericum perforatum* L. (DSB36) Clusiaceae	1	1	2	1	5	+
*Laurus nobilis* L. (DSB38) Lauraceae	1	1	2	1	5	−
*Lavandula dentata* L. (DSB40) Lamiaceae	0	2	2	1	5	+
*Micromeria juliana* (L.) Benth. ex Rchb. (DSB47) Lamiaceae	1	1	2	1	5	−
*Parietaria judaica* L. (DSB49) Urticaceae	2	2	0	1	5	−
*Rosa canina* L. (DSB55) Rosaceae	1	2	1	1	5	+
*Scrophularia nodosa* L. (DSB59) Scrophulariaceae	1	1	2	1	5	−
*Silene italica* (L.) Pers. s.l. (DSB60) Caryophyllaceae	2	1	1	1	5	−
*Sonchus asper* (L.) Hill subsp. *glaucescens* (Jord.) Ball (DSB64) Asteraceae	2	1	1	1	5	−
*Taraxacum officinale* Weber (DSB67) Asteraceae	1	2	1	1	5	−
*Verbascum thapsus* L. subsp. *thapsus* (DSB73) Scrophulariaceae	1	2	2	0	5	−
*Achillea millefolium* L. subsp. *millefolium* (DSB1) Asteraceae	1	1	1	1	4	−
*Asplenium onopteris* L. (DSB8) Aspleniaceae	1	1	1	1	4	−
*Asplenium trichomanes* L. s.l. (DSB9) Aspleniaceae	1	1	1	1	4	−
*Borago officinalis* L. (DSB11) Boraginaceae	1	1	1	1	4	−
*Capparis spinosa* L. subsp. *rupestris* (Sm.) Nyman (DSB15) Capparaceae	1	1	1	1	4	−
*Cichorium intybus* L. s.l. (DSB18) Asteraceae	0	1	2	1	4	−
*Equisetum hyemale* L. (DSB26) Equisetaceae	0	0	3	1	4	+++
*Fumaria capreolata* L. subsp. *capreolata* (DSB30) Fumariaceae	1	1	1	1	4	−
*Lavatera cretica* L. (DSB41) Malvaceae	1	1	1	1	4	−
*Ligustrum vulgare* (DSB42) Oleaceae	1	1	1	1	4	−
*Phyllitis scolopendrium* (L.) Newman subsp. *scolopendrium *(DSB50) Aspleniaceae	1	1	1	1	4	++
*Smilax aspera* L. (DSB63) Smilacaceae	1	1	1	1	4	−
*Stachys sylvatica* L. (DSB65) Lamiaceae	0	1	2	1	4	−
*Stellaria media* (L.) Vill. s.l. (DSB66) Caryophyllaceae	1	1	1	1	4	−
*Teucrium fruticans* L. subsp. *fruticans* (DSB68) Lamiaceae	1	0	2	1	4	−
*Urtica membranacea* Poir. ex Savigny (DSB70) Urticaceae	1	1	1	1	4	−
*Valeriana officinalis* L. (DSB71) Valerianaceae	1	1	2	0	4	+
*Viburnum tinus* L. subsp. *tinus* (DSB74) Caprifoliaceae	1	1	1	1	4	−
*Zea mays* L. (DSB75) Poaceae	1	1	1	1	4	−
*Centranthus ruber* (L.) DC. subsp. *rubber* (DSB16) Valerianaceae	1	1	0	1	3	−
*Cymbalaria muralis* Gaertn., B. Mey. et Scherb s.l. (DSB21) Scrophulariaceae	1	0	1	1	3	−
*Linaria vulgaris* Mill. subsp. *vulgaris* (DSB43) Scrophulariaceae	1	1	0	1	3	−
*Silene latifolia *Poir. subsp. *alba* (Mill.) Greuter et Burdet (DSB61) Caryophyllaceae	0	1	1	1	3	−
*Silene vulgaris* (Moench) Garcke s.l (DSB62) Caryophyllaceae	0	1	1	1	3	−
*Gentiana cruciata* L. s.l. (DSB31) Gentianaceae	0	1	1	0	2	−
*Robinia pseudoacacia* L. (DSB54) Fabaceae	1	0	0	1	2	−
*Dittrichia viscosa* (L.) Greuter s.l. (DSB24) Asteraceae	1	0	0	0	1	−
*Lolium rigidum* Gaudin subsp. *rigidum* (DSB44) Poaceae	0	0	0	1	1	−
*Ranunculus millefoliatus* Vahl (DSB53) Ranunculaceae	0	0	0	1	1	−

The last column refers to antimicrobial activity. Active against one (+), two (++), and three or more (+++) bacterial strains.

**Table 2:** Antibacterial activity of plant extracts towards *S. mutans, S. sobrinus, A. viscosus, and L. casei. *

	Solvent	*S. sobrinus *			*S. mutans *			*A. viscosus *			*L. casei *		
		A	B	C	A	B	C	A	B	C	A	B	C
*Cotinus coggygria *	W	11,8 ± 0,4	14,3 ± 0,5	16 ± 0		9 ± 0	10 ± 0		10,1 ± 0,3	11 ± 0		12 ± 0	16,8 ± 0,3
	M			9,3 ± 0,4			10,1 ± 0,3			9,3 ± 0,4			
	H		10,8 ± 0,4	11,8 ± 0,4									
*Equisetum hyemale *	M			10 ± 0			13 ± 0					13,1 ± 0,3	15,8 ± 0,4
*Juniperus communis *	H		10,1 ± 0,3	11,8 ± 0,4	9 ± 0	10,1 ± 0,3	13,1 ± 0,3	10 ± 0	10,1 ± 0,3	11,8 ± 0,4			
*Helichrysum litoreum *	M					9 ± 0	11,8 ± 0,4	10,2 ± 0,4	11,8 ± 0,4	18 ± 0			
*Phyllitis scolopendrium *	M				11,8 ± 0,4	14,3 ± 0,5	16 ± 0				11,8 ± 0,4	15 ± 0	17 ± 0
*Bellis perennis *	M	10,1 ± 0,3	12 ± 0	15,8 ± 0,4									10,8 ± 0,4
*Ceterach officinarum *	M		9,1 ± 0,3	11,8 ± 0,4									10 ± 0
*Thymus vulgaris *	M		11,8 ± 0,4	14 ± 0						13,1 ± 0,3			
*Cupressus sempervirens *	M									9,3 ± 0,4			10,1 ± 0,3
*Lavandula angustifolia *	M			11,8 ± 0,4									12 ± 0
*Gentiana lutea *	M							9 ± 0	11,8 ± 0,4	14,3 ± 0,5			
*Rosa canina *	M					11,8 ± 0,4	14,7 ± 0,5						
*Artemisia annua *	M									10 ± 0			
*Cynoglossum creticum *	M												13 ± 0
*Eucalyptus globulus *	M			10 ± 0									
*Hypericum perforatum *	W			10,8 ± 0,4									
*Lavandula dentata *	M			13 ± 0									
*Sambucus nigra *	M									11,8 ± 0,4			
*Valeriana officinalis *	W			10,1 ± 0,3									
*Verbena officinalis *	W			10,1 ± 0,3									
Triclosan 0.3%		15 ± 0			16 ± 0			20 ± 0			17 ± 0		

Antibacterial activity is expressed as mean (±standard deviation) zones of inhibition (mm). A = 12.5 mg/mL, B = 25 mg/mL, C = 50 mg/mL. W = water, E = ethanol, H = hexane.

**Table 3:** MIC and MBC of more effective plant extracts against *S. mutans*, *S. sobrinus*, *A. viscosus*, and *L. casei. *

		*S. sobrinus *		*S. mutans *		*A. viscosus *		*L. casei *	
		MIC90	MBC	MIC90	MBC	MIC90	MBC	MIC90	MBC
*Cotinus coggygria *	W	12.5	50	50	100	50	100	50	100
	M	25	100	50	100	25	100	100	100
	H	50	100	>100	>100	>100	>100	>100	>100
*Equisetum hyemale *	M	100	100	>100	>100	>100	>100	>100	>100
*Juniperus communis *	H	>100	>100	>100	>100	>100	>100	>100	>100
*Helichrysum litoreum*	M	>100	>100	5	25	25	50	>100	>100
*Phyllitis scolopendrium *	M	>100	>100	>100	>100	>100	>100	>100	>100

Minimum inhibitory concentration (MIC) and minimum bactericidal concentration (MBC) are expressed in mg/mL. W = water, E = ethanol, H = hexane.
